# Drivers of heterogeneity in synovial fibroblasts in rheumatoid arthritis

**DOI:** 10.1038/s41590-023-01527-9

**Published:** 2023-06-05

**Authors:** Melanie H. Smith, Vianne R. Gao, Preethi K. Periyakoil, Alejandro Kochen, Edward F. DiCarlo, Susan M. Goodman, Thomas M. Norman, Laura T. Donlin, Christina S. Leslie, Alexander Y. Rudensky

**Affiliations:** 1 Division of Rheumatology, Department of Medicine, Hospital for Special Surgery, New York, NY USA; 2 Howard Hughes Medical Institute and Immunology Program at Sloan Kettering Institute, Ludwig Center for Cancer Immunotherapy, Memorial Sloan Kettering Cancer Center, New York, NY USA; 3 Computational and Systems Biology Program, Memorial Sloan Kettering Cancer Center, New York, NY USA; 4 Weill Cornell Medical College and Graduate School, New York, NY USA; 5 Arthritis and Tissue Degeneration Program and the David Z. Rosensweig Genomics Research Center, Hospital for Special Surgery, New York, NY USA; 6 Department of Pathology and Laboratory Medicine, Hospital for Special Surgery, New York, NY USA

**Keywords:** Autoimmunity, Rheumatoid arthritis, Next-generation sequencing, Chronic inflammation

## Abstract

Inflammation of non-barrier immunologically quiescent tissues is associated with a massive influx of blood-borne innate and adaptive immune cells. Cues from the latter are likely to alter and expand activated states of the resident cells. However, local communications between immigrant and resident cell types in human inflammatory disease remain poorly understood. Here, we explored drivers of fibroblast-like synoviocyte (FLS) heterogeneity in inflamed joints of patients with rheumatoid arthritis using paired single-cell RNA and ATAC sequencing, multiplexed imaging and spatial transcriptomics along with in vitro modeling of cell-extrinsic factor signaling. These analyses suggest that local exposures to myeloid and T cell-derived cytokines, TNF, IFN-γ, IL-1β or lack thereof, drive four distinct FLS states some of which closely resemble fibroblast states in other disease-affected tissues including skin and colon. Our results highlight a role for concurrent, spatially distributed cytokine signaling within the inflamed synovium.

## Abstract (web summary)

Smith et al. present a resource detailing drivers of transcriptional heterogeneity of synovial fibroblasts cell states in the inflamed joints of human patients with rheumatoid arthritis.

## Main

Rheumatoid arthritis (RA), a systemic autoimmune disease with predominantly articular manifestations, is characterized by hyperplasia of both the synovial lining, which interfaces the synovial fluid-filled joint space, as well as the synovial sublining, which exhibits increased vascularization and an influx of leukocytes. Both the lining and sublining fibroblast-like synoviocytes (FLS) undergo proliferation and activation, assuming states in which they stimulate angiogenesis, produce proinflammatory cytokines and chemokines and invade adjacent articular cartilage and bone^1^. Expression of major histocompatibility complex (MHC) class II molecules by activated FLS is associated with synovial inflammation^2,3^ and correlates with disease activity^4^. HLA-DR^+^ FLS expression of soluble mediators, including the proinflammatory cytokines interleukin (IL)-6 and IL-15 and chemokines CCL2, CXCL9 and CXCL12 along with adhesion molecules such as ICAM1 and VCAM1 suggests interactions with leukocytes^2^. In support of this possibility, previous in vitro studies have shown that HLA-DR^+^ FLS are capable of presenting antigens to CD4^+^ T cells^5–7^. Furthermore, production of the aforementioned proinflammatory chemokines by FLS likely acts as a feedforward mechanism to further facilitate recruitment of diverse immune cell types. Indeed, recent studies of the cellular makeup of synovial tissue from patients with RA using single-cell RNA sequencing (scRNA-seq) identified a diverse mix of migratory and resident cells of hematopoietic and non-hematopoietic origin including different CD4^+^ and CD8^+^ T cell subsets, myeloid cells and FLS^2,8,9^. These observations suggest that FLS activation states in the RA synovium are likely driven by a diversity of infiltrating innate and adaptive immune cells and that this modulation ultimately impacts disease pathogenesis.

Thus, we sought to undertake an in-depth investigation of the spectrum of FLS states in the inflamed RA synovium as well as the drivers underlying the observed heterogeneity through paired scRNA and assay for transposase-accessible chromatin with sequencing (scRNA/ATAC-seq) and in vitro modeling of FLS transcriptional responses to key immune cell-derived proinflammatory cytokines. We then mapped the spatial distribution of FLS heterogeneity and transcriptional responses by employing spatial transcriptomic (ST) analyses and multiplex imaging. Our findings suggest that spatially constrained FLS responses to three leukocyte-derived cytokines, tumor necrosis factor (TNF), interferon (IFN)-γ and IL-1β, or lack thereof, drive the formation of four distinct FLS states in the inflamed RA synovium.

## Results

### Synovial inflammation is associated with expansion of activated FLS states

**Fig. 1:**
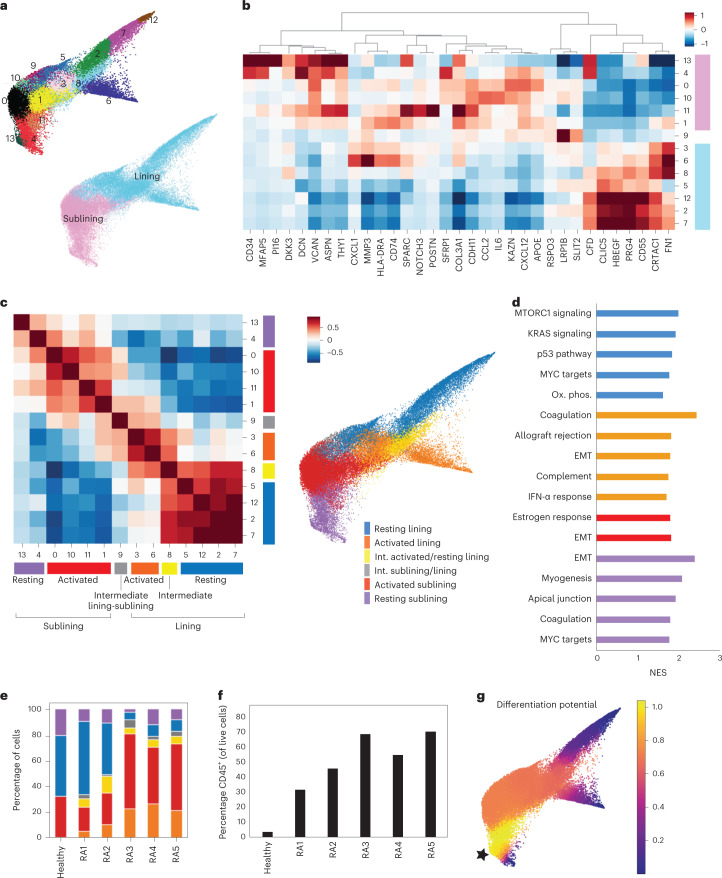
FLS states in the RA synovium exhibit evidence of activation by immune cells. **a**, Force-directed layout of 14 FLS clusters identified by scRNA-seq analysis with Harmony batch correction with corresponding annotations of synovial localization. **b**, Heat map of selected differentially expressed genes (DEGs) for each cluster colored by synovial localization. **c**, Cluster-by-cluster correlation of the mean expression of highly variable genes in clusters from **a** with defined FLS states colored on the force-directed layout. **d**, GSEA showing top HALLMARK pathways with false discovery rate (FDR) < 0.1 (up to 5) for each of the states defined in **c**. EMT, epithelial–mesenchymal transition; NES, normalized enrichment score. **e**, Percent FLS in each state defined in **c** for each synovial tissue sample. **f**, Percent CD45^+^ cells in each of the dissociated synovial tissue samples by flow cytometry. **g**, Trajectory analysis using Palantir starting from a sublining cell from the healthy synovial sample (starting point marked with a star).

**Extended Data Fig. 1:**
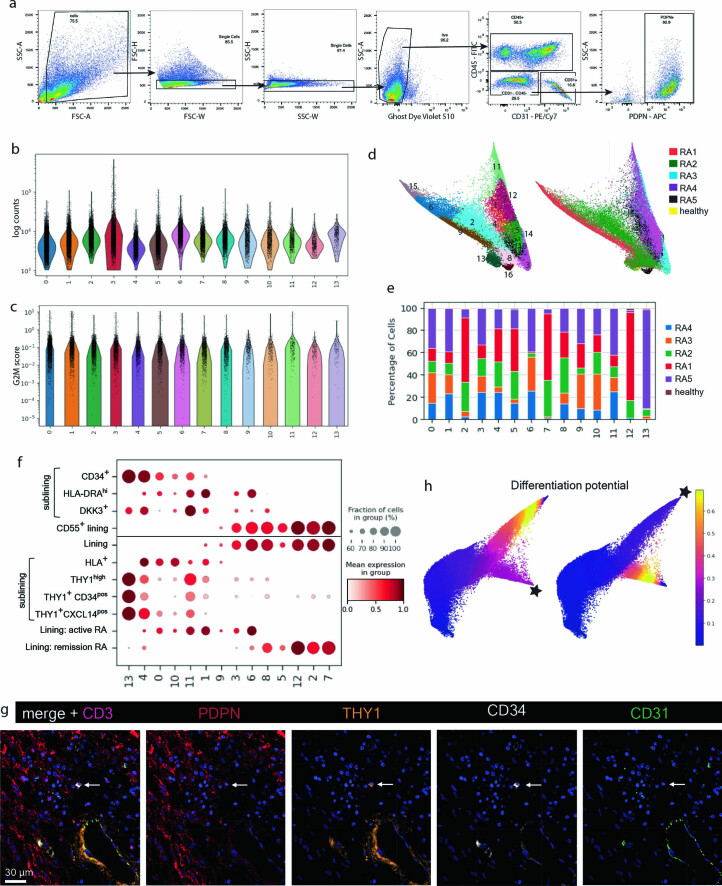
scRNA-seq analysis of FLS isolated from RA synovium. **a**, Gating strategy for FACS sorting of FLS to exclude PDPN^-^ mural cells. Numbers below population are percentage of parent population. **b**, Numbers of UMI counts per cluster. **c**, Cell cycle G2M score per cluster. **d**, Force directed layout without batch correction colored by cluster (left) and patient (right). **e**, Cluster composition by patient after Harmony batch correction. **f**, Dotplot showing the relative per cluster expression of previously published cluster-derived gene signatures: Zhang et al^2^ above and Alivernini et al^4^ below horizontal line. **g**, Representative confocal image of PDPN (red), THY1 (orange), CD34 (white), CD31 (green), CD3 (magenta) and nuclear marker (blue) from RA synovial tissue (*n* = 4 tissues). White arrow indicates individual CD34^+^ THY1^+^PDPN^low^CD31^−^ cell. **h**, Trajectory analysis using Palantir starting from cells in either the activated (left) or resting (right) lining states (starting point marked with a star).

To test the possibility that joint inflammation in patients with RA leads to a marked expansion of cytokine-activated FLS, we sought to characterize FLS transcriptomes and chromatin accessibility at a single-cell resolution for RA tissues with varying degrees of inflammatory infiltration. All patients had established seropositive (CCP^+^) RA that met the 2010 American College of Rheumatology/European League Against Rheumatism classification criteria^10^ and were not treated with any biologic within the preceding 3 months. All samples were histologically classified as containing lymphocyte aggregates as seen in the lympho-myeloid pathotype^11^, but had varying degrees of lymphocytic infiltration and a range of Krenn scores^12^. Fluorescence-activated cell sorting (FACS)-sorted FLS (CD45^−^CD31^−^PDPN^+^) were isolated from five patients with RA and one healthy control and subjected to paired scRNA/ATAC-seq using the 10x Multiome platform (patients RA1–RA5 in Supplementary Table 1 and Extended Data Fig. 1a). After extensive filtering and Harmony batch correction^13^ of the scRNA-seq data, we obtained 14 clusters with 36,719 FLS (Fig. 1a, Extended Data Fig. 1b–e and Supplementary Table 2). Using established markers^2,4,14^, some of which are shown in Fig. 1b, we identified lining and sublining clusters as well as one intermediate cluster (9). Our extensive dataset included FLS subsets previously identified from both active and remission RA^2,4^ (Extended Data Fig. 1f). Notably, both with and without batch correction, the lining FLS formed two distinct groups of clusters distinguished by expression of *FN1*, *MMP3* and *HLA-DR* versus *PRG4*, *CLIC5* and *CD55*— a distinction associated with active RA versus remission, respectively.

While each cluster had distinct transcriptomic signatures, such as *NOTCH3* expression in cluster 11 marking perivascular FLS^15^, gene set enrichment analysis (GSEA) highlighted shared functionality between clusters (Supplementary Table 3). In fact, assessing cluster-by-cluster correlations, defined four FLS states with distinct inferred functionality and two clusters (8 and 9) that formed intermediates between states (Fig. 1c,d and Supplementary Table 4). In each synovial compartment (lining and sublining) there was an ‘activated’ state associated with inflammatory responses and cytokine signaling and a contrasting state lacking this signature that we termed ‘resting’. This differentiation between ‘activated’ and ‘resting’ was not only based on the pathway analysis, but also on accessible motifs identified by ATAC-seq and the presence or absence of FLS-specific cytokine response gene signature expression as will be discussed subsequently. The identified resting lining FLS state shared a transcriptional profile with lining FLS from patients with RA in remission^4^ and was associated with multiple pathways involved in cell growth and proliferation (Fig. 1d). In addition, resting lining FLS were characterized by high expression of genes involved in the production of synovial fluid and extracellular matrix (ECM) (*XYLT1*, *ITGB8* and *PRG4*) and axonal guidance (*SEMA5A*, *ANK3*, *ROBO2*, *NTN4* and *UNC5C*), likely reflecting their unique function in the synovium. The activated lining FLS state, marked by elevated HLA-DR expression, displayed both IFN-α and IFN-γ responses as well as additional inflammatory gene expression signatures such as the complement pathway. The activated sublining FLS clusters (0, 1, 10 and 11) were enriched for cytokine responses, most notably TNF and IFN-γ. The resting sublining FLS state expressed genes associated with ECM homeostasis and mitogenic pathways. Of note, both the sublining and activated lining FLS states expressed genes associated with epithelial–mesenchymal transition (EMT) signaling (*DCN*, *VCAN*, *FBLN1*, *IGFBP4*, *FBN1*, *MFAP5*, *SFRP1*, *COL1A1*, *TGFBR3*, *MMP2* and *LAMA2*) likely related to FLS function as mobile, ECM-secreting stromal cells.

Within the resting sublining FLS state, we observed that cluster 13 exhibited features of progenitor cells including the highest level of CD34 expression. Notably, the gene expression features of cluster 13 showed extensive similarity to those of PI16^+^ ‘universal fibroblasts’ identified across tissues in a perturbed-state fibroblast atlas^16^. As tissue progenitors or ‘stem-like’ cells can be frequently found as aggregates within specialized anatomical niches commonly associated with vasculature, we explored spatial distribution of these CD34^high^THY1^+^PDPN^+^ FLS using immunofluorescence (IF) confocal microscopy. Counter to our expectations, we found them dispersed as solitary cells throughout the inflamed synovium without conspicuous association with the vascular endothelium (Extended Data Fig. 1g). This finding suggests a possibility that in the inflamed RA synovium, FLS regenerative capacity is preserved in a non-compartmentalized manner.

To better understand how the degree of lymphocytic infiltration affects the FLS states, we assessed the relative abundance of FLS states across synovial samples (Fig. 1e). In the tissues with a larger percentage of CD45^+^ leukocytes, there was an expansion of the activated sublining and activated lining states as well as contraction of the resting lining state (Fig. 1f). The percentage of resting sublining FLS was relatively constant, further highlighting a potential progenitor-like role for these FLS, whose differentiation potential requires further study. Of note, even the healthy synovium contained activated sublining FLS leading us to speculate that these may have yet to be defined homeostatic functions. The correlation between the proportion of activated FLS states and leukocytic infiltration suggested that the states may be driven by interactions between cell types and are likely fluid.

Given that we suspected FLS activation states to be transient in response to interactions with locally infiltrating leukocytes, we performed a trajectory analysis using Palantir^17^ to better understand the relationship between FLS states (Fig. 1g). Starting from cells in the resting sublining of the healthy synovial sample, there were end points in both the activated and resting lining, but not the activated sublining indicating that the latter may be an intermediate state. However, when starting from the resting or activated lining, there was little differentiation potential (Extended Data Fig. 1h). This supports the notion that FLS can differentiate from the sublining to the lining, but not in reverse. The lack of end points in the activated sublining is consistent with previous analyses showing that CXCL12^+^ FLS were intermediates in a RA FLS trajectory^18^. Additional research is needed to fully understand human FLS differentiation.

### Shared features of FLS states across diseases

**Fig. 2:**
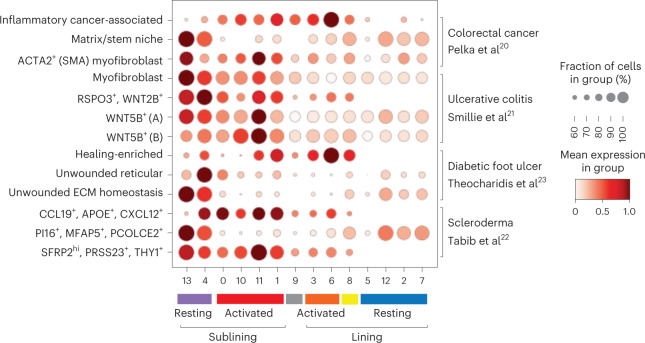
Shared functional gene expression programs in FLS and non-synovial fibroblasts across tissues and diseases. Dot-plot showing relative expression of selected gene signatures from published tissue fibroblast populations^20–23^ in FLS clusters colored according to FLS states.

**Extended Data Fig. 2:**
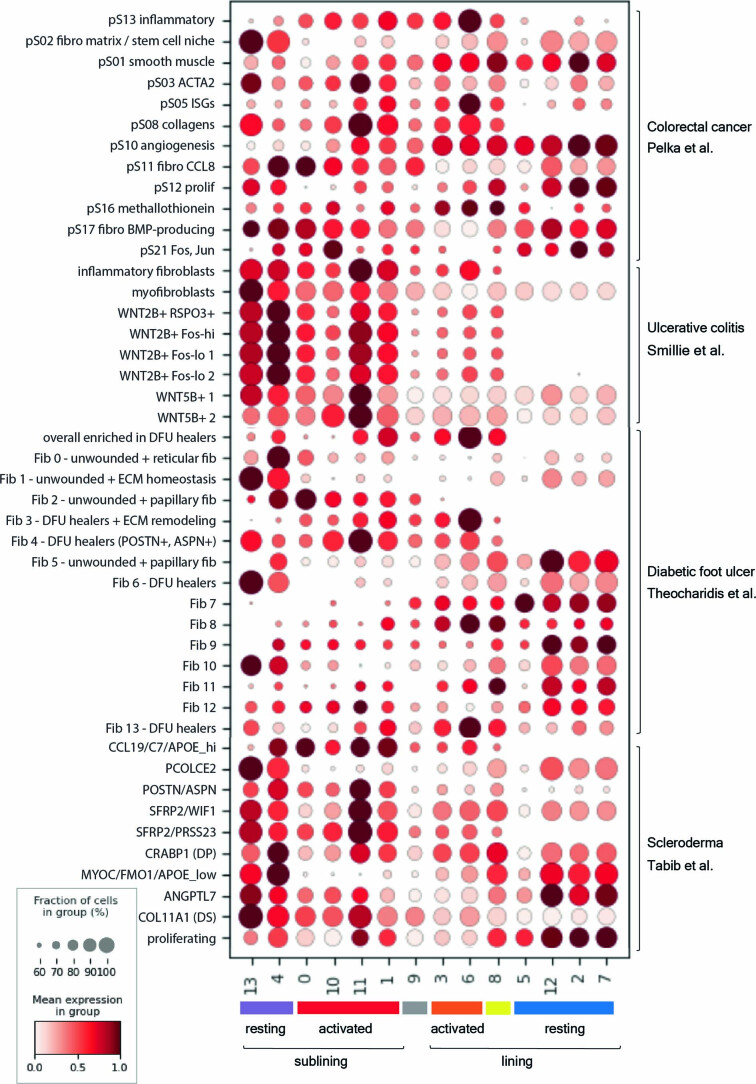
Expression of non-synovial fibroblast gene signatures in FLS. Dot plot showing relative expression of all gene signatures from published tissue fibroblast populations^20–23^ in FLS clusters colored according to FLS states. Abbreviations: ISG: interferon- stimulated genes; DFU: diabetic foot ulcers; ECM: extracellular matrix; DP: dermal papilla; DS: dermal sheath.

Previous cell population-based studies suggested distinct diversity of transcriptional features of human fibroblasts in different anatomical locations and heritable imprinting of their ‘topography’^19^. However, our observation of conserved ‘universal fibroblast’ features of PI16^+^ FLS suggested that there might be an overlap between disease-induced states of anatomically distinct tissue fibroblasts affected by different pathologies. Thus, we next sought to explore whether the FLS states we identified were unique to the synovium or RA-associated inflammation, or alternatively, were shared with fibroblast populations observed in other diseases and tissues (Fig. 2 and Extended Data Fig. 2). For this comparative analysis, we took advantage of several recent scRNA-seq datasets of colonic^20,21^ and dermal fibroblasts^22,23^, in which at least five fibroblast clusters were delineated.

As expected from the aforementioned presence of the ‘universal fibroblast signature’, our cluster 13 FLS shared transcriptional similarity with fibroblasts from both the colon and skin. In the colon, this transcriptional signature was observed in two fibroblast populations implicated in creating an intestinal stem cell niche: ECM-producing fibroblasts and myofibroblasts. In the skin, this transcriptional signature was observed in fibroblasts from unwounded skin responsible for ECM homeostasis and in a fibroblast population that undergoes contraction in scleroderma as compared to healthy skin (PI16^+^MFAP5^+^PCOLCE2^+^).

Unexpectedly, we observed a transcriptional similarity between inflammatory cancer-associated fibroblasts in colorectal cancer and the activated lining FLS state. The latter FLS state was also similar to transcriptional states of fibroblasts in diabetic foot ulcers that were able to successfully heal. In contrast, activated sublining FLS were transcriptionally similar to ACTA2^+^ myofibroblasts and WNT5B^+^ fibroblasts in the colon as well as two populations of dermal fibroblasts expanded in scleroderma as compared to healthy skin (CCL19^+^APOE^+^CXCL12^+^ and SFRP2^hi^PRSS23^+^THY1^+^). Thus, inflammation-induced perturbations in the overall composition of the FLS population and spectrum of FLS states were shared with fibroblasts found in other tissues in a range of pathologies.

### FLS states exhibit distinct transcriptional regulation

**Fig. 3:**
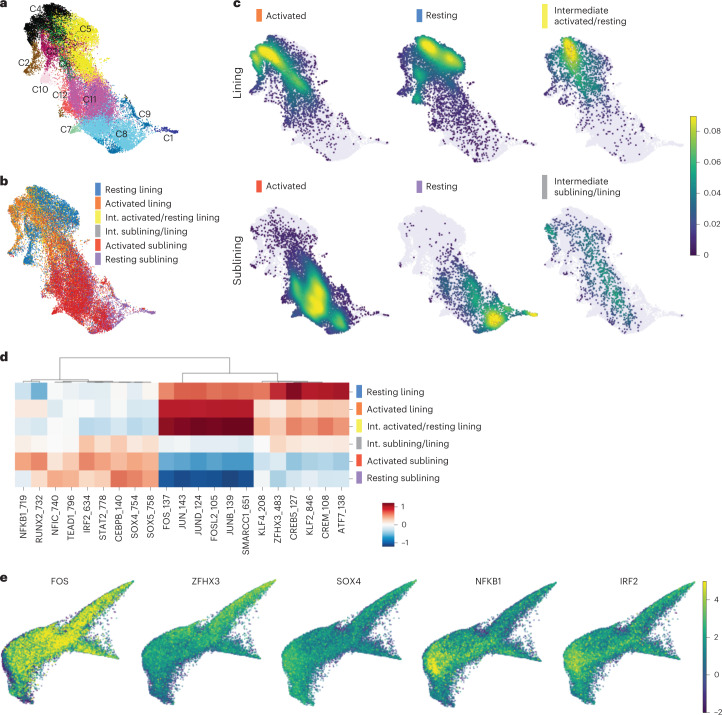
Chromatin accessibility analysis of FLS states reveals their distinct transcriptional regulation. **a**, UMAP of 12 FLS clusters identified by tile-based scATAC-seq analysis after Harmony batch correction. **b**, Annotations of FLS states on the scATAC-seq UMAP. **c**, Projection of FLS states onto scATAC-seq UMAP. **d**, Heat map with top-six differentially accessible TF motifs identified by ChromVAR for each FLS state. Motifs filtered to include only those for which the corresponding TF was expressed by >20% of cells in the corresponding state. **e**, ChromVAR *z* scores projected onto scRNA-seq force-directed layout for a selection of top differentially accessible TF motifs derived from each FLS state.

**Extended Data Fig. 3:**
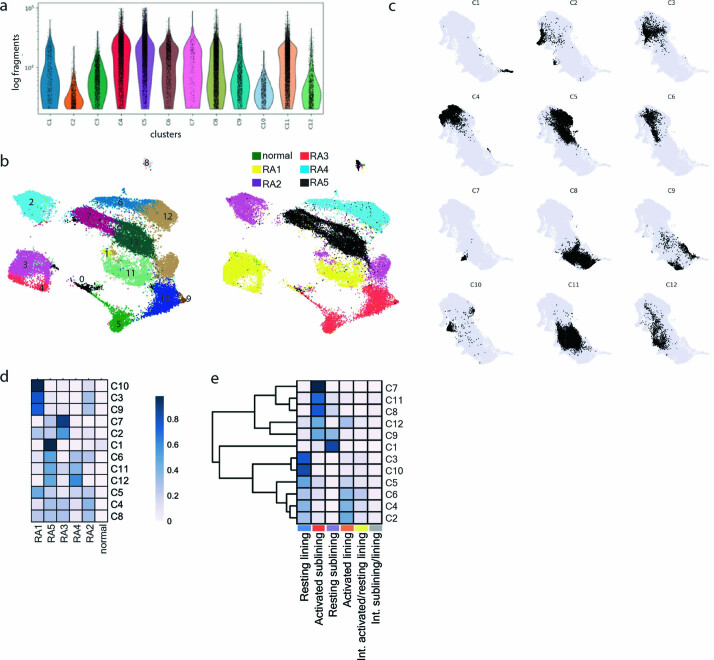
Paired scATAC-seq analysis of isolated FLS. **a**, Number of fragments detected in each scATAC-seq cluster from Fig. 3a. **b**, scATAC-seq UMAP without batch correction colored by clusters (left) and patients (right). **c**, Individual scATAC-seq clusters imposed on scATAC-seq UMAP after Harmony batch correction. **d**, Contribution of each patient to scATAC-seq clusters. **e**, scATAC-seq cluster composition by FLS states.

To gain insights into the transcription factors (TFs) and upstream signaling pathways, which may regulate the observed FLS states, we analyzed paired scATAC-seq datasets. Unsupervised clustering and Harmony batch correction^13^ resulted in 12 clusters with 30,963 FLS encompassing lining and sublining FLS (Fig. 3a and Extended Data Fig. 3a–d). Distinct FLS states identified by scRNA-seq analyses occupied divergent areas of the scATAC-seq Uniform Manifold Approximation and Projection (UMAP) (Fig. 3b,c) partially overlapping with the identified scATAC-seq clusters (Extended Data Fig. 3e). To infer differential TF activity in identified FLS states, we performed chromatin accessibility variation analysis using chromVAR^24^. For this analysis, we used the paired scRNA-seq data as a filter to assess only motifs for the TF families whose members were expressed by >20% of cells in the corresponding state. We observed marked differences in enrichment of distinct TF binding motifs within open chromatin sites with differential motif accessibility between states (Fig. 3d,e and Supplementary Table 5). The activated FLS state was enriched for accessibility of AP-1 TF family motifs (FOS, JUN, JUNB, JUND and FOSL2), whose increased contribution to gene regulation downstream of fibroblast growth factor and immune receptor signaling, such as IL-1R1, has been suggested to play a role in tissue-destructive properties of FLS in RA^25,26^. Open chromatin sites characteristic of the activated sublining FLS state were enriched for IRF, STAT and NF-κB family motifs implicating a distinct set of inflammatory pathways, such as IFN and TNF signaling in establishing this state. Contrary to these two major types of inflammatory activation, the resting lining FLS state was distinguished by the accessibility of motifs of homeobox TF family members (for example, ZFHX3), which besides serving as major regulators of tissue development and organization, including the joint-specific origins of FLS^27^, control fibroblast quiescence (for example, PRRX1)^28,29^. In support of a role for homeobox TFs in modulating FLS activation, homeobox binding sites were identified as potential repressors of transcriptional responses to TNF stimulation in FLS^30^ and homeobox family member CUX1 has specifically been shown to bind to NF-κB and alter its activity as reflected by either downregulation^31^ or upregulation^32^ of specific NF-κB-regulated cytokines and chemokines. Consistent with their role in producing components of the synovial fluid such as lubricin (*PRG4*), the most differentially accessible TF motif in the resting lining FLS state was CREB5, which has been shown to be necessary for *PRG4* expression in articular cartilage^33^. Finally, the resting sublining FLS were distinguished by accessible *cis*-regulatory elements enriched for motifs of SOX and TEAD family members, which play a role in maintaining quiescent undifferentiated states in stem cells and early progenitors, as well as NFIC, which has been implicated in the regulation of transforming growth factor (TGF)-β signaling in both tooth^34^ and hair follicle^35^ development. These results suggest that distinct states of FLS in the inflamed RA joint were dependent upon local stimulation by immune cell-derived factors, foremost proinflammatory cytokines or avoidance of these inflammatory exposures.

### Cytokine signaling drives transcriptional heterogeneity

**Fig. 4:**
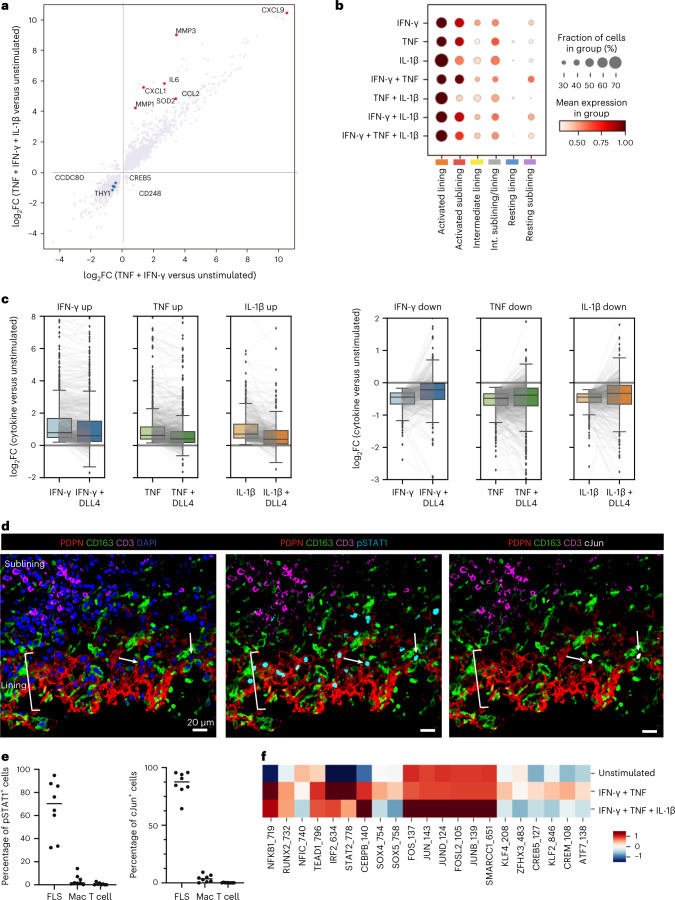
Cytokine signaling drives transcriptional FLS heterogeneity. **a**, Changes in gene expression (log fold change) after combinatorial stimulation of cultured FLS by the cytokines indicated. Red and blue dots highlight upregulated and downregulated genes, respectively. **b**, Dot-plot showing relative expression of the identified cytokine response signatures in each of the FLS states. **c**, Effect of Notch signaling on FLS cytokine responses. Cultured FLS were treated with the individual cytokines indicated in vitro and RNA-seq was used to identify genes that were upregulated (left) or downregulated (right). Box plots compare the distribution of log_2_ fold changes in the expression of these genes (in stimulated versus control) in FLS treated with each cytokine alone or in combination with DLL4. Gray lines connect individual genes across conditions. Boxes show lower and upper quartiles of the data with a line marking the median. Whiskers indicate extent of data, capped at 1.5 times the interquartile range, outside of which points are marked as outliers (ticks). **d**, Representative confocal images of staining for pSTAT1 (cyan), cJun (white), PDPN (red), CD163 (green), CD3 (magenta) and nuclear marker (blue) (*n* = 4 tissues with total of eight sections). White arrows indicate FLS with nuclear cJun staining in the lining. **e**, Percentage of cells staining for nuclear pSTAT1 or cJun annotated as FLS, macrophage (Mac) or T cell as identified by cell markers PDPN (FLS), CD163 (macrophage) and CD3 (T cell) in confocal images from **d**. **f**, ChromVAR *z* score of motifs from Fig. 3d in cultured FLS that were unstimulated, simulated with TNF and IFN-γ or stimulated with TNF, IFN-γ and IL-1β. Source data

**Extended Data Fig. 4:**
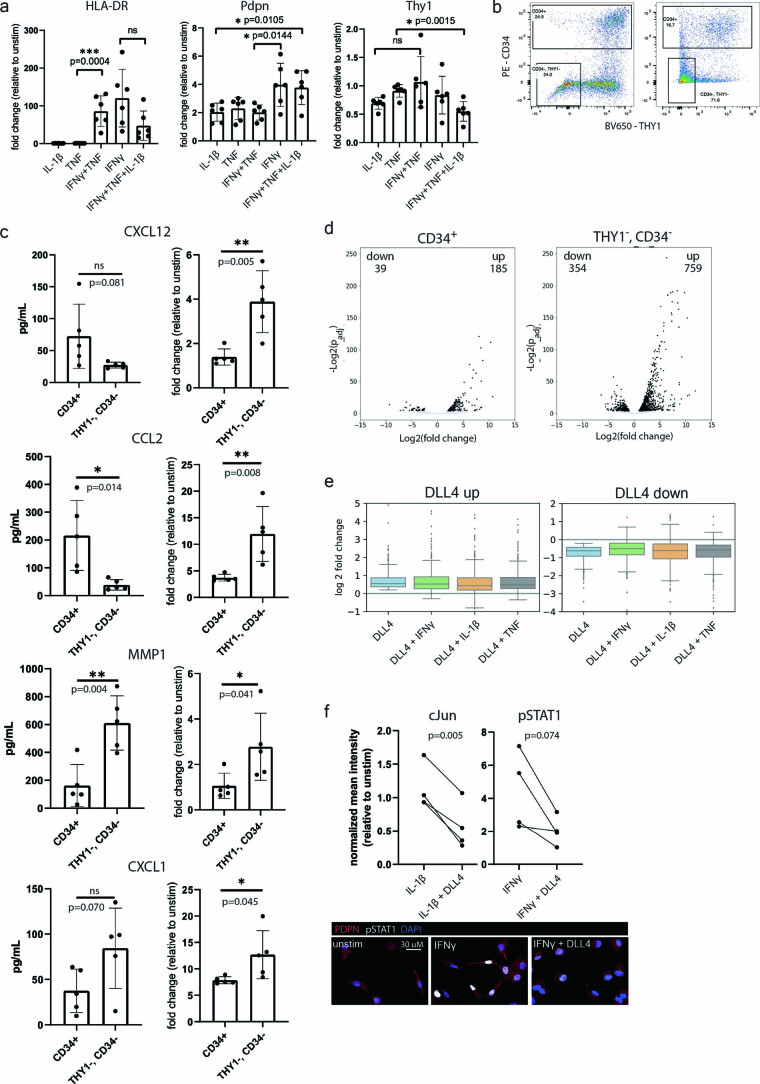
Effects of cytokine and Notch signaling on FLS. **a**, Surface protein expression of HLA-DR, PDPN and Thy1 on cultured FLS as measured by flow cytometry (mean fluorescence intensity) after indicated stimuli relative to unstimulated FLS (*n* = 6: FLS isolated from 6 different tissues and assayed independently). Error bars: mean ± standard deviation. Statistical comparisons made via unpaired t-test with two-tailed p-value. **b**, Two representative flow cytometry plots from tissues with different population distributions showing gating strategy for FACS sorting of CD34^+^ and THY1^-^CD34^-^ FLS used following the gating strategy shown in Extended Data Fig. 1a. **c**, Expression of soluble proteins by CD34^+^ sublining or THY1^-^CD34^-^ lining FLS directly sorted from dissociated synovium and cultured for 24 h either without stimulation (left) or with TNF, IFNγ and IL-1β (right) (*n* = 5 tissues). Error bars: mean ± standard deviation. Statistical comparisons made via unpaired t-test with two-tailed p-value. **d**, Changes in gene expression by bulk RNA-seq in sorted CD34^+^ sublining or THY1^-^CD34^-^ lining FLS stimulated with TNF, IFNγ and IL-1β relative to unstimulated controls (*n* = 2 of tissues used in panel **c**). Differentially expressed genes were determined using DEseq2 with cutoff at p < 0.05 and absolute log2FC > 1. **e**, Effect of the cytokine simulation in combination with DLL4 on genes induced or downregulated by FLS treatment with DLL4 alone. Box plots compare the distribution of log2 fold changes in the expression of these genes (in stimulated versus control) in FLS treated with DLL4 alone or in combination with cytokine. Boxes show lower and upper quartiles of the data with line marking the median. Whiskers indicate extent of data, capped at 1.5 times the interquartile range, outside of which points are marked as outliers (ticks). **f**, Effect of Notch signaling on transcription factor activation in cultured FLS as measured by IF. Mean normalized intensity of nuclear cJun or pSTAT1 after simulation with IL-1β or IFNγ, respectively, with or without DLL4 relative to unstimulated (*n* = FLS from 4 tissues). Statistical comparisons made via paired t-test with two- tailed p-value. Below: representative confocal images of cultured FLS stimulated with IFNγ with or without DLL4 and stained for pSTAT1 (cyan), PDPN (red) and nuclear marker (blue). Source data

To test the above possibility and to elucidate influences of inflammatory factors on transcriptomes of FLS states we sought to deconvolute their complex transcriptomes by establishing cell-type-specific cytokine-induced programs. For identification of immune cell-derived cytokine transcriptional responses and their contributions to distinct features of FLS state-specific transcriptomes, we employed in vitro stimulation of cultured FLS, known to rapidly lose their phenotypic heterogeneity with subsequent passages^15^, by candidate proinflammatory cytokines and other factors. For these experiments, FLS were isolated from four RA synovial tissue samples and cultured for three passages before pooling FLS from all donors and performing the stimulations in triplicate. FLS were stimulated with three major cytokines implicated in RA (TNF, IFN-γ and IL-1β, either individually or in combination) and the resulting gene expression changes were assessed using RNA-seq (Fig. 4a and Supplementary Table 6). These cytokine stimulations, particularly in combination, had a dramatic effect on cell morphology as well as cell surface protein expression (Extended Data Fig. 4a). We found that in vitro stimulation of FLS with both TNF- and IFN-γ-induced expression of genes, including *CCL2* and *IL6*, which were highly expressed by ex vivo isolated activated sublining FLS (Fig. 4a and clusters 10 and 0). On the other hand, genes whose expression was suppressed in response to these cytokines (for example, *CCDC80* and *CD248* in Fig. 4a) were highly upregulated by CD34^hi^THY1^+^PI16^+^ FLS (cluster 13) suggesting that these FLS in the resting sublining are shielded from exposure to inflammatory mediators and that these cytokines may even lead to the loss of this state. Interestingly, these CD34^hi^ cells also seem to be less responsive to cytokine stimulation. When we sorted CD34^+^ sublining and THY1^−^CD34^−^ lining FLS directly from dissociated synovial tissue and stimulated them with the triple combination of TNF, IFN-γ and IL-1β, the CD34^+^ FLS did not increase their production of soluble mediators to the same degree as the THY1^−^CD34^−^ lining FLS, including for proteins such as CCL2 and CXCL12, which are typically produced in the sublining and expected to be preferentially expressed by CD34^+^ FLS versus lining FLS (Extended Data Fig. 4b,c). We additionally measured changes in gene expression in these same cells and found that the CD34^+^ FLS had fewer significantly up- or downregulated genes as compared to the THY1^−^CD34^−^ FLS (Extended Data Fig. 4d). In the same vein, *CREB5*, which is upregulated in the resting lining FLS state, was also downregulated in response to in vitro stimulation with TNF and IFN-γ suggesting that besides the resting FLS in the sublining, resting lining FLS also seem to be spared from the full-scale effects of inflammatory cytokines. Finally, genes induced in FLS subjected to in vitro stimulation by the combination of TNF, IFN-γ and IL-1β, which included *MMP3* and *CXCL1*, were most differentially expressed in ex vivo isolated activated lining FLS.

Mapping of cytokine response gene signatures established by the above in vitro analyses onto our scRNA-seq datasets revealed the most pronounced expression of the FLS-specific cytokine response gene signatures in the activated lining FLS state (Fig. 4b). In contrast, the dominant cytokine response signatures in the activated sublining FLS state were associated with a dual TNF and IFN-γ stimulation. Notably, the resting states were nearly devoid of cytokine response gene signature expression.

Notch signaling induced by ligands expressed by the vascular endothelium has been suggested to factor prominently in the differentiation of perivascular and sublining FLS in the RA synovium^15^. This raised the question as to whether Notch signaling can potentially modulate transcriptional responses of FLS to proinflammatory cytokines within the sublining. To explore this possibility, we investigated changes in gene expression induced in FLS upon stimulation with TNF, IFN-γ and IL-1β in the presence or absence of plate-bound Notch ligand Delta like-4 (DLL4). This analysis revealed a global dampening of transcriptional responses to all three cytokines (Fig. 4c and Supplementary Table 7): for both up- and downregulated genes, the observed changes were blunted across the board. Notch signaling simulated by DLL4 seemed to be the dominant signaling pathway as the addition of cytokines did not affect the expression of genes that were up- or downregulated by DLL4 (Extended Data Fig. 4e). We validated this finding using IF-based imaging of TFs cJun or phosphorylated STAT1 (pSTAT1) in cultured FLS stimulated with IL-1β or IFN-γ, respectively, either alone or in the presence of DLL4 and found that DLL4 decreased the mean nuclear fluorescence intensity of both TFs in response to cytokine stimulation (Extended Data Fig. 4f). This finding was unexpected considering that previous studies suggested that Notch signaling augments macrophage responses to TLR ligands and increases production of proinflammatory cytokines^36^. However, there may be multiple specific regulatory mechanisms in play as previous reports also show that IFN-γ can inhibit Notch signaling in macrophages^37^. These results raise an intriguing question as to whether coincident engagement of inflammatory and developmental signaling pathways may result in different functional outcomes depending on a given cell type.

**Extended Data Fig. 5:**
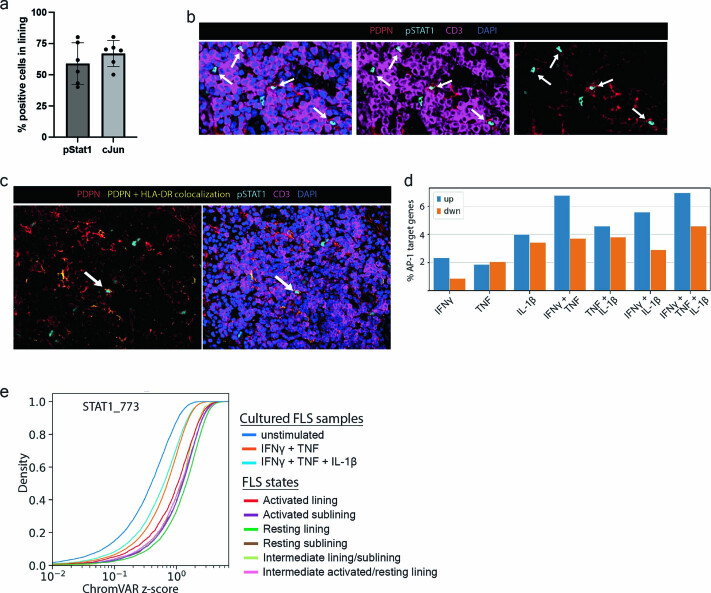
Evidence of STAT1 and AP-1 activation in FLS. **a**, Percentage of cells staining for nuclear pSTAT1 or cJun found within the synovial lining, which was manually annotated based on tissue architecture, in confocal images from Fig. 4d (*n* = 3 tissues, 6 sections). Error bars: mean ± standard deviation. **b**, Representative confocal images of a lymphocyte aggregate within the sublining stained for phosphorylated STAT1 (cyan), PDPN (red), CD3 (magenta) and nuclear marker (blue) (*n* = 4 tissues, 8 sections). White arrows indicate FLS with nuclear pSTAT1. **c**, Representative confocal images of a lymphocyte aggregate stained for the antigens in (**b**) above as well as the colocalization of HLA-DR and PDPN (yellow) as defined by pixels with fluorescence intensity in the top 10% for both HLA-DR and PDPN (*n* = 3 tissues). **d**, Percent of genes identified in the multiome dataset with accessible AP-1 motifs in their promoters that were up- or down-regulated in cultured FLS stimulated with the indicated cytokines. AP-1 target genes (1024 total) in the activated lining FLS state were identified as those with motif matches with a log-odds score of at least 10 (computed by motifmatchr). **e**, Dynamics of chromatin accessibility of the STAT1 motif containing cis-regulatory elements across ex vivo FLS states and in vitro cytokine stimulated or unstimulated FLS. Empirical cumulative distribution function (ECDF) 600 plots of ChromVAR z-scores for STAT1 motif from scATAC-seq analysis of the indicated cultured FLS samples or FLS states are shown. Source data

We next looked for evidence of cytokine signaling in the synovial lining through IF-assisted imaging of activated (nuclear) TFs STAT1 and cJun, key downstream targets of IFN and IL-1β signaling, respectively. We identified numerous pSTAT1^+^ FLS in the synovial lining and found that some of these cells also stained for nuclear cJun (Fig. 4d). Whereas cJun^+^ FLS were primarily identified in or near the synovial lining (Extended Data Fig. 5a), pSTAT1^+^ FLS were also identified within T cell aggregates found in the sublining (Extended Data Fig. 5b). Of note, some of the pSTAT1^+^ FLS also express HLA-DR (Extended Data Fig. 5c) consistent with a well-recognized role of IFN-γ in driving MHC class II expression. Remarkably, the majority of both cJun^+^ or pSTAT1^+^ cells in the synovium were FLS highlighting that these stromal cells represent major cytokine-signaling targets in the synovium (Fig. 4e).

To further validate the effect of cytokine stimulation on regulation of gene expression, we isolated FLS from two of the same RA synovial tissue samples subjected to scATAC/RNA-seq (RA4 and RA5 from Supplementary Table 1), stimulated them at passage 3 with TNF, IFN-γ and IL-1β or TNF and IFN-γ and performed scATAC/RNA-seq. This allowed us to cross-reference the observed TF motifs at modulated chromatin accessibility sites with cytokine simulation to those observed in the FLS states revealed by scATAC-seq analyses of ex vivo isolated cells (Fig. 4f). We found that stimulation of FLS with a combination of TNF and IFN-γ resulted in enrichment of IRF, STAT and NF-κB family TF motifs at differentially accessible chromatin sites in comparison to unstimulated FLS, closely matching those in the activated sublining state of FLS ex vivo. In contrast, a triple combination of TNF, IFN-γ and IL-1β resulted in differential chromatin remodeling at sites enriched for AP-1 TF family motifs. The latter observation was consistent with a previous report of remodeling of chromatin regions containing NF-κB and AP-1 binding motifs in response to IL-1β^38^. Notably, the differentially accessible sites enriched for AP-1 family motifs in the FLS stimulated with the triple combination of cytokines (Fig. 4f) were nearly identical to those observed in the ex vivo isolated activated lining FLS state (Fig. 3d). In addition, expressed genes containing AP-1 motifs in their accessible promoter sites that we identified in lining FLS in our multiome dataset were induced by cytokine stimulation of FLS in vitro — particularly by the triple combination of TNF, IFN-γ and IL-1β (Extended Data Fig. 5d). A comparison of cytokine-stimulated samples to the unstimulated control (Extended Data Fig. 5e) offered an explanation for the seemingly unexpected decrease in accessibility of *cis*-regulatory elements containing STAT motifs in the activated lining FLS (Fig. 3d) despite the presence of a robust IFN-γ response gene expression signature (Fig. 4b) and STAT1 phosphorylation (Fig. 4d). This observation was most likely due to a relative decrease in STAT accessibility of a subset of these elements caused by combined IL-1β, IFN-γ and TNF exposure, whereas the corresponding transcript levels were not markedly impacted. Together, these analyses of transcriptomes and epigenomes strongly support a role of combinatorial stimulation by TNF and IFN-γ in facilitating the establishment of the activated sublining FLS state and a triple combination of TNF, IFN-γ and IL-1β in establishing the activated lining FLS state.

### FLS states and cytokine signaling are spatially constrained

**Fig. 5:**
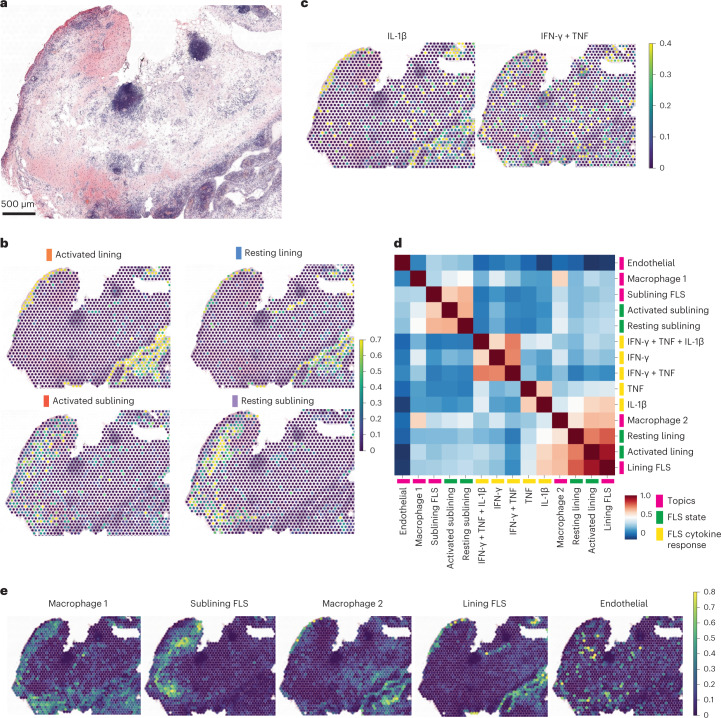
Cytokine signaling is spatially constrained and correlated with cellular localization. **a**, H&E staining of a tissue section used for ST (RA3 in Supplementary Table 1) (*n* = 2 tissues for ST; other tissue shown in Extended Data Fig. 6). **b**, Relative expression of FLS states in each RNA capture area on the ST slide. **c**, Relative expression of selected FLS cytokine response signatures in each RNA capture area on the ST slide. **d**, Correlation between FLS state gene signatures derived from scRNA-seq data, in vitro cytokine response gene signatures and cell types as defined by topic modeling within individual RNA capture spots for RA3. **e**, Expression of cell type-specific topics from **d** in each RNA capture area on the ST slide.

**Extended Data Fig. 6:**
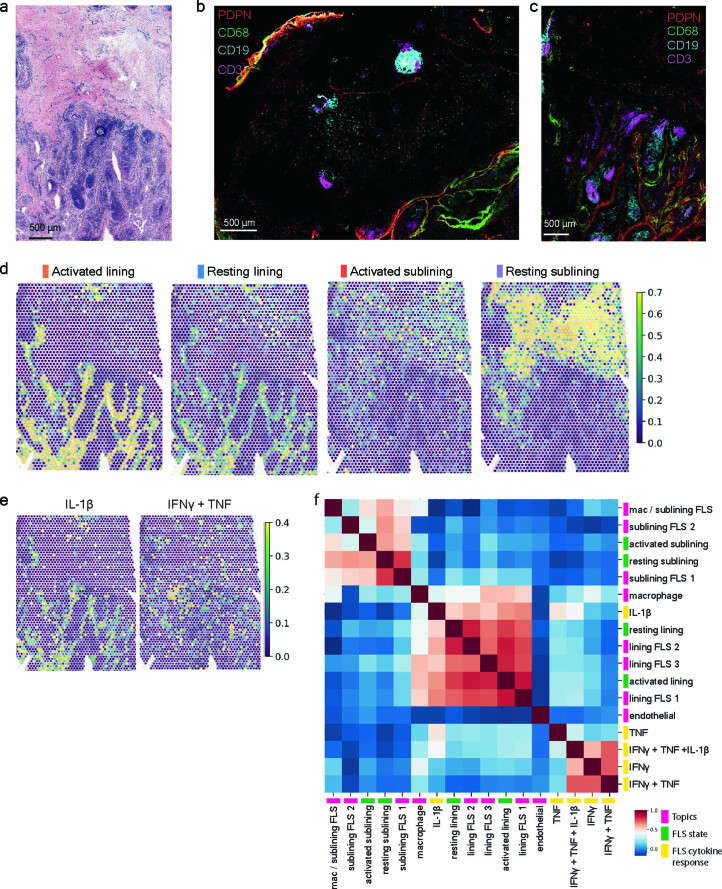
Spatial transcriptomic analysis of inflamed RA synovial tissue. **a**, H&E staining of second tissue section used for ST (RA6 in Supplementary Table 1) (*n* = 2 tissues for ST). **b, c**, IF images of serial tissue sections directly adjacent to those used for ST analysis for RA3 (**b**) and RA6 (**c**) stained for PDPN (red), CD68 (green), CD19 (cyan) and CD3 (magenta). (*n* = 2 tissues, 4 sections adjacent to those used for ST). **d**, Relative expression of gene signatures of FLS states in each RNA capture area on the ST slide. **e**, Relative expression of selected FLS cytokine response gene signatures in each RNA capture area on the ST slide. **f**, Correlation between FLS state gene signatures derived from scRNA-seq data, in vitro cytokine response gene signatures (select ones shown in e) and cell types as defined by topic modeling within individual RNA capture spots for RA6.

The observations above suggest that distinct FLS states in the inflamed synovium are established in a spatial manner as the result of locally produced inflammatory cytokines from distinct types of immune cells invading the RA synovium. To define the spatial distribution of FLS states within the RA synovium, we performed ST analyses using the 10x Visium platform combined with multiplex IF imaging of adjacent tissue sections for two additional inflamed RA synovial samples (RA3 and RA6 in Supplementary Table 1). The hematoxylin and eosin (H&E) staining of the sections subjected to ST analysis showed prominent lymphocyte aggregates as well as copious synovial lining (Fig. 5a and Extended Data Fig. 6a). IF analysis showed scattered PDPN^+^ FLS and CD68^+^ macrophages as well as lymphocyte aggregates (CD3^+^ and CD19^+^ cells) in the sublining region and multiple regions of lining populated by FLS and macrophages (Extended Data Fig. 6b,c). The ST datasets were integrated with our scRNA-seq analyses to map the transcriptional signatures from the four FLS states. This showed that the resting and activated FLS seemed to intermix without well-defined regions in both the lining and sublining (Fig. 5b). We next applied the in vitro FLS-specific cytokine response gene signatures to spatial gene expression maps. We found that the IL-1β response signature mapped predominantly to the lining compartment, whereas response signatures for TNF, IFN-γ and their combination were more scattered (Fig. 5c). As multiple cells could contribute to each RNA capture spot, we confirmed that the IL-1β response gene signature observed in ST analysis was contributed by FLS by creating a modified IL-1β response gene signature that only contained genes uniquely expressed by FLS based on recent scRNA-seq analysis of RA synovium^2^.

**Extended Data Fig. 7:**
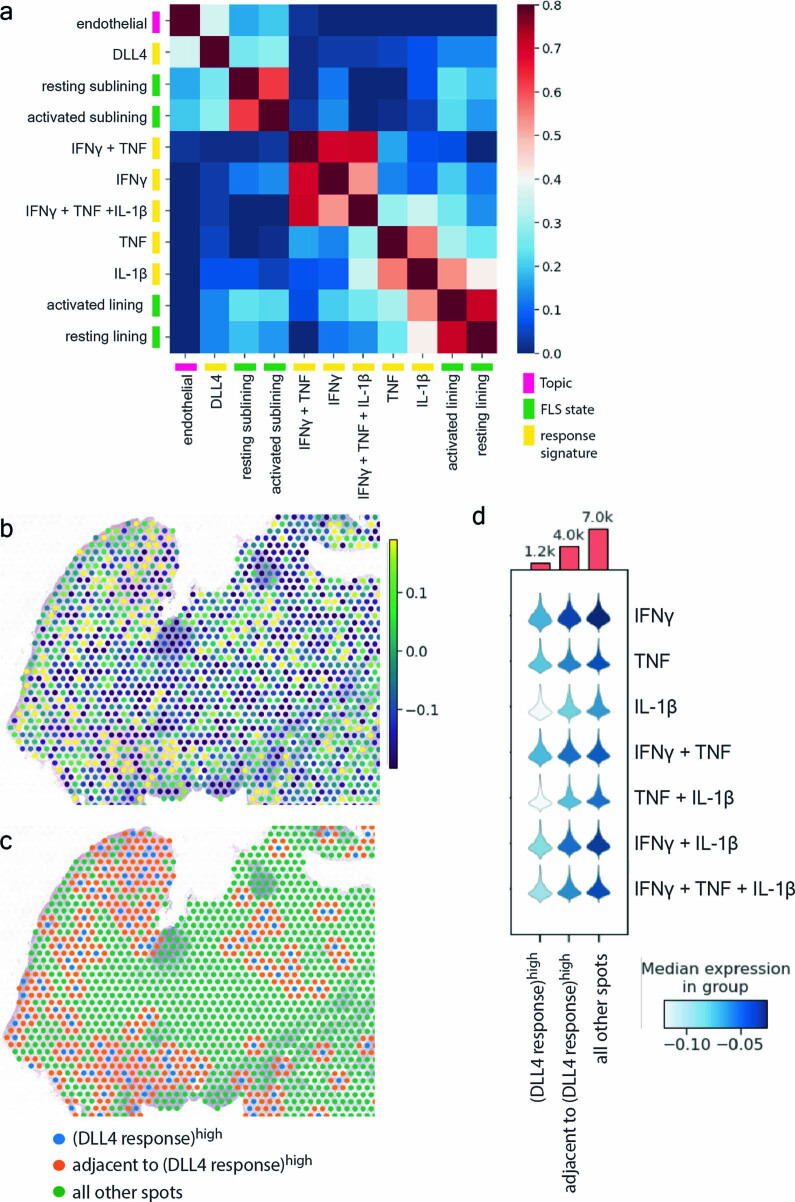
Spatial distribution of Notch signaling in FLS. **a**, Correlation between localization of endothelial cells (defined by topic modeling), FLS state gene signatures derived from scRNA-seq data and in vitro cytokine or DLL4 response gene signatures within individual RNA capture spots (*n* = 2 sections for patient RA3 in Supplementary Table 1). **b**, Relative expression of the FLS response gene signature to stimulation by plate bound DLL4 in each RNA capture area on the ST slide (*n* = 2 tissues, 2 sections each: RA3 shown). **c**, Annotation of each RNA capture area into three groups: DLL4 response gene signature score in the top 10^th^ percentile (blue), directly adjacent to these areas (orange), or all other spots (green) (*n* = 2 tissues, 2 sections each: RA3 shown). **d**, Violin plot showing the expression of the FLS-specific cytokine response gene signatures in the areas defined in panel (**c**) (aggregated data from all 4 sections from 2 tissues: RA3 and RA6). The top shows the number of total RNA capture areas in the groups defined in panel (**c**) for all tissue sections.

To further characterize synovial microenvironments, we applied topic modeling to the ST datasets to identify specific cell types within each spot and colocalize these with our FLS states and cytokine response gene signatures (Fig. 5d,e and Supplementary Table 8). An unbiased correlation of the FLS states, cytokine response gene signatures and topic-defined cell types showed a colocalization of S100A8^+^ tissue macrophages, lining FLS and the IL-1β response gene signature. Similar analyses of an independent RA sample confirmed these results (Extended Data Fig. 6d–f). This suggests that IL-1β derived from either resident macrophages that have taken on an inflammatory program or infiltrating activated monocytes play a role in lining FLS activation. Additionally, we found that the expression of the gene signature derived from FLS stimulated in vitro with Notch ligand DLL4 co-localized with both the endothelial topic and the sublining FLS states (Extended Data Fig. 7a). Consistent with our in vitro results (Fig. 4c and Extended Data Fig. 4f), areas with high expression of the DLL4 response gene signature and to a lesser extent those areas immediately adjacent, were characterized by a dampening of cytokine response gene signature expression (Extended Data Fig. 7b–d). These results suggest that cytokine and developmental signaling pathways shape multiple spatially distinct microenvironments within the inflamed RA synovium.

## Discussion

Phenotypic and functional heterogeneity of stromal cells within a given tissue depends on both constant cell-intrinsic differentiation programs and cell-extrinsic cues afforded by stable and transient interactions with tissue-resident and infiltrating cells. The latter are represented for the most part by diverse types of immune cells, which, when activated, produce cytokines and other mediators that can act on the stromal cells changing the range of their physiological or homeostatic states. In RA, the synovium, which in health is a non-barrier immunologically quiescent tissue, experiences a massive influx of both innate and adaptive immune cells. In this setting, FLS experience differentiation signals, such as the endothelium-derived Notch signaling that shapes FLS in the synovial sublining region^15^ and coincident exposures to multiple cytokines and other inflammatory mediators. Using paired scRNA/ATAC-seq and ST analyses assisted by in vitro generated cytokine response signatures, we demonstrated that leukocyte-derived cytokines play a key role in the formation of discrete, spatially defined, yet likely dynamic FLS states with distinct inferred functionality that differ between the sublining and lining.

Perhaps the most notable results were the enrichment of genes downstream of IFN-γ, TNF and IL-1β in the activated lining FLS state, which was also associated with AP-1 activation. In addition, lining FLS accounted for the overwhelming majority of synovial STAT1 and Jun activation. These were unexpected findings given that most synovial lymphocytes, which have been shown to express proinflammatory cytokines^2^, are found in the sublining near the vasculature. We also observed that, contrary to a reported Notch-mediate potentiation of inflammatory cytokine responses in macrophages^36^, Notch activation attenuated cytokine responses in FLS. This finding may explain the relatively dampened cytokine response gene signatures observed in the activated sublining FLS state, which was distinguished by the highest *NOTCH3* expression, as compared to the activated lining FLS state. The even more pronounced dampening of cytokine response gene signatures observed in the resting sublining FLS state is likely because CD34^+^ FLS, which are transcriptionally similar to progenitor fibroblasts identified in other human tissues, are intrinsically less responsive to cytokine stimulation than lining FLS. Future studies will help determine the role of this population in the synovium in both health and disease.

While it is likely that the combination of IFN-γ and TNF that drive the activated sublining FLS state originate from activated T cells within lymphocyte aggregates, the source(s) of cytokines within the lining microenvironment is less clear. The observation that the activated lining FLS transcriptome was enriched for genes downstream of IFN-γ, TNF and IL-1β is consistent with the expression of the latter two cytokines by synovial macrophages^2^ as well as the coincident positioning of inflammatory tissue macrophages with the local IL-1β signature in the synovial lining in our ST analysis. Potential sources of IFN-γ and STAT1 activation within the synovial lining remain unclear. While CD8^+^ T cells have been described as the dominant producers of IFN-γ within the synovium, it is possible that other cells such as natural killer cells^3^ or myeloid cells^39^ may contribute. Finally, it is possible that the prominent pSTAT1 signal observed in the synovial lining FLS (Fig. 4d), which has also been reported previously^40^, is the result of the action of alternative drivers of STAT1 activation including type I IFN^40^, whose expression can be driven by IL-1β^41^.

The prominent IL-1β response signature in the cytokine-activated lining FLS state is notable given its possible functional and therapeutic implications. First, IL-1β is the primary inducer of matrix metalloproteinases (MMPs), which have been implicated in FLS invasiveness^42^. This invites the possibility of a functional dichotomy between sublining and lining FLS in RA parallel to that observed in an experimental arthritis model in mice, where the lining FLS are uniquely responsible for destruction of cartilage and bone^43^. Blocking IL-1 may antagonize the capacity of FLS to assume this MMP-expressing lining state and thus it is possible that for a subset of patients the addition of IL-1 inhibition during flares could prove effective for preventing joint destruction. Second, the activated lining FLS state may drive migration of neutrophils into the synovial fluid, where there is a surfeit of neutrophils during RA flares. In this regard, the inflammatory cancer-associated fibroblasts in colorectal adenocarcinomas, which we found to share extensive transcriptional similarity with the activated lining FLS state, express neutrophil chemoattractants including CXCL1 and CXCL8 and their location was spatially correlated with the accumulation of neutrophils^20^. In RA, IL-1β produced by macrophages in the synovial lining could drive the expression of CXCL1 observed in the activated lining FLS state. Finally, the expansion of the activated lining FLS state observed in highly inflamed RA synovium may have prognostic implications. A recent study of pathotypes in inflammatory bowel disease showed an association of an IL-1β-activated fibroblast signature with a lack of response to multiple therapies^44^.

In conclusion, we established a spatial atlas of heterogeneity of synovial fibroblast states in RA defined by their distinct transcriptional signatures and patterns of chromatin accessibility driven by differential local exposure to immune cell-derived proinflammatory cytokines. Notably, the cytokine milieu and FLS activation states differ in the lining and sublining arguing that we need to better understand these microenvironments and the functional implications of their differences. Further definition of synovial microenvironments guided by the framework established in this study is warranted considering the pronounced synovial heterogeneity observed in RA and the fact that our multiome datasets were exclusively derived from tissue samples of CCP+ patients treated with conventional synthetic disease-modifying anti-rheumatic drugs. The resulting datasets will serve as a rich resource for future investigation of RA pathogenesis through integration of unique and shared characteristics of inflamed synovial fibroblasts described herein with other disease-associated signals, such as complement activation^45^ and potential antigen presentation via HLA-DR.

## Methods

### Human synovial tissue

RA synovial tissue was obtained from patients enrolled in the Hospital for Special Surgery (HSS) FLARE study of patients with RA undergoing arthroplasty or synovectomy (approved by HSS IRB no. 2014-233). The healthy synovial tissue was obtained from Memorial Sloan Kettering Cancer Center (MSKCC) (approved by IRB no. 06-107). Enrollment complied with all relevant ethical regulations of both institutions. Informed consent was obtained from all participants. All materials and data transferred between HSS and MSKCC were covered under material transfer agreements. On the day of surgery, samples were cryopreserved as small fragments in CryoStor CS10 (Stem Cell Technologies, 07959). RA synovial tissue quality and grading of synovitis^12^ were evaluated by histological analysis (H&E).

### Sample preparation for single-cell sequencing

Synovial tissue samples were disaggregated into a single-cell suspension as described previously^2^. Briefly, fragments were minced and enzymatically digested (Liberase TL (Sigma-Aldrich) 100 μg ml^−1^ and DNase I (New England Biolabs) 100 μg ml^−1^ in RPMI) for 30 min at 37 °C. Disaggregated cells were assessed for viability (Nexcelom Cellometer Auto 2000) and then stained with antibodies to CD45 (2D1), CD31 (WM59), PDPN (NZ-1.3) and Ghost Dye Violet 510 (Tonbo) for fluorescence-activated cell sorting (BD FACSAria III Cell Sorter) (Supplementary Table 9 lists antibodies used in this study). Synovial fibroblasts (CD45^−^, CD31^−^ and PDPN^+^) were collected and individual nuclei were prepared using the 10x Genomics protocol CG000365 Rev A. Nuclei were submitted for sequencing via Chromium Single Cell Multiome ATAC+ Gene Expression (10x Genomics) by the Integrated Genomics Operation (IGO) core facility at the MSKCC.

The IGO stained single nuclei suspensions with Trypan blue and counted using the Countess II Automated Cell Counter (Thermo Fisher). Following quality control, nuclei were incubated with transposition mix for 60 min at 37 °C and then loaded onto Chromium Next GEM Chip J (10x Genomics PN 1000234) and GEM generation of 10,000 nuclei proceeded using the Chromium Next GEM Single Cell Multiome ATAC+ Gene Expression Reagent Bundle (10x Genomics PN 1000283) according to the manufacturer’s protocol. After reverse transcription, both DNA and complementary DNA fragments were pre-amplified with seven cycles of PCR. ATAC libraries were constructed using the Single Index kit N (10x Genomics PN 1000212) and seven cycles of PCR and pooled equimolar for sequencing on a NovaSeq 6000 in a PE50/49 run using the NovaSeq 6000 SP Reagent kit (100 cycles) (Illumina). An average of 240 million paired reads was generated per sample. Full-length cDNA was amplified for an additional six cycles before library preparation proceeded according to 10x Genomics protocols with 10–12 cycles of PCR. Gene expression libraries were sequenced in a PE28/88 run on a NovaSeq 6000 with the NovaSeq 6000 S2 Reagent kit (100 cycles) (Illumina). Each sample received 217 million reads on average.

For cultured cytokine-stimulated FLS, synovial tissues were dissociated into single cells as above, cultured in MEM α (Thermo Fisher Scientific Gibco, 12561056) with 10% fetal bovine serum (R&D systems, S11550) as well as 1% penicillin/streptomycin (Thermo Fisher Scientific, 15070063) and 1% l-glutamine (Thermo Fisher Scientific, 25030081). Cells were passaged using TrypLE Express Enzyme (Thermo Fisher Scientific Gibco, 12605010) until an FLS monoculture was present (≥3 passages). At passage 4, FLS were stimulated with cytokines as concentrations similar to those used in multiple studies in the literature (TNF (20 ng ml^−1^) + IFN-γ (5 ng ml^−1^) or TNF (20 ng ml^−1^) + IFN-γ (5 ng ml^−1^) + IL-1β (1 ng ml^−1^)) for 24 h before collecting and isolating nuclei as above (TNF (PeproTech, 300-01A), IFN-γ (Roche, 11040596001), IL-1β (PeproTech, 200-01B)).

### Preprocessing of single-cell multiome ATAC+ gene expression data

RNA and ATAC libraries for each patient were aligned using CellRanger-arc software (v.1.0.0, 10x Genomics) against 10x genomics reference refdata-cellranger-arc-GRCh38-2020-A using default parameters. The output files fragments.tsv.gz and filtered_feature_matrix.h5 were utilized for downstream processing and quality control. We then performed additional cell filtering steps: (1) cells with a high fraction of mitochondrial molecules were filtered (>20%); (2) cells with low library size were filtered (<1,024 molecules); (3) two clusters resembling contaminating immune cell populations were removed. Putative doublets were removed using the DoubletDetection package (10.5281/zenodo.2658729). Cells or nuclei that passed these quality control cutoffs were used to generate sparse count matrices and filtered fragments.tsv.gz files for downstream analysis.

### Single-cell RNA-seq data analysis

#### Preprocessing, dimensionality reduction and clustering

Combining the six patient samples yielded a filtered count matrix of 36,719 cells by 36,391 genes, with a median of 6,189 molecules per cell. The count matrix was then normalized by library size and scaled to 100,000 per cell for analysis of the combined dataset. Highly variable genes were identified using the scanpy highly_variable_genes function with batch_key = ‘sample’. Ribosomal genes, mitochondrial genes and MALAT1 were masked from downstream analysis. Principal-component analysis (PCA) was performed on the normalized expression of highly variable genes with the top 50 principal components (PCs) retained. We next performed batch correction using scanpy’s harmony_integrate function with the PCs described above as the basis, resulting in batch-corrected PCs for all downstream analysis. We next performed clustering on the combined dataset using Phenograph with *k* = 100 to identify 14 clusters. We tested the ‘stableness’ of clustering assignments as *k* varies from 10 to 500 by computing the adjusted Rand index using sklearn’s adjusted_rand_score function and found that clustering was stable around *k* = 100. To aid subtype annotation, we merged these clusters into meta-clusters based on the correlation in cluster mean expression of highly variable genes.

#### Visualization of scRNA-seq

We used force-directed layout projections to generate lower dimensional representations using fa2 from the ForceAtlas2 package. Specifically, we use the top 30 batch-corrected PCs, edgeWeightInfluence = 0.8, jitterTolerance = 1 and gravity = 1.

#### Differential expression in scRNA-seq

We performed differential expression for the following comparisons (1) each fibroblast state versus rest and (2) each unsupervised cluster versus rest (Supplementary Tables 2 and 4). All differential expression was performed using MAST (v.1.8.2)^46^, which provides a flexible framework for fitting a hierarchical generalized linear model to the expression data. We used a regression model that adjusts for cellular detection rate (cngeneson, or number of genes detected per sample):

$${\rm{Y}}\_{\rm{i}},{\rm{j}}\backsim {\rm{condition}}+{\rm{cngeneson}}$$

where condition represents the condition of interest and Y_i,j is the expression level of gene i in cells in cluster j, transformed by natural logarithm with a pseudocount of 1. We considered genes to be significantly differentially expressed for Bonferroni-adjusted *P* value < 0.05.

#### Identifying enriched gene pathways in scRNA-seq data

Enriched gene pathways were identified using pre-ranked GSEA, as implemented by the R package fGSEA^47^ using 2,000 permutations. Gene ranks were calculated using −log(*P* value) × log fold change based on MAST^46^ differential expression. To assess enriched pathways in clusters, we used HALLMARK and KEGG subsets of Canonical Pathways in MSigDB v.7.1 (ref. ^48^). We considered pathways with Benjamini–Hochberg-adjusted *P* values < 0.25 to be significant.

#### scRNA-seq pseudotime trajectory inference

Pseudotime trajectories were generated using Palantir^17^. A starting cell for the trajectory (ID TAGTGTGGTGGAAACG) was identified by choosing a cell at the maximal point of the normal sample in the force-directed layout projection. The terminal cells were automatically identified by palantir. We used default parameters except for num_waypoints = 1,000.

#### Scoring gene signature expressions

We first transformed the library size-normalized, log-transformed data by *z* score and calculated the average expression of each curated gene set per cell type subtracted from the average expression of a reference set of genes using the score_genes function in scanpy. The subsequent cell type scores were transformed again by *z* score. For comparisons to published datasets, we used the top 30 genes after sorting by adjusted *P* value (*P*adj) for top DEGs for each unsupervised cluster/cell type.

#### Correlating spatial gene signature expression

We computed the Pearson correlation coefficient of their expressions across individual spots for all samples combined. The signatures were generated as described above.

### Single-cell ATAC-seq data analysis

#### Preprocessing, dimensionality reduction, clustering

We preprocessed the filtered fragments.tsv file using the ArchR package^49^ v.1.0.1. Specifically, we binarized sparse accessibility matrices binned at 500-bp tiles across the genome. Cells with fewer than 2,000 fragments and TSS < 4 are filtered, as well as cells that did not pass the scRNA-seq filtering step. We then performed iterative latent semantic indexing on the tile matrix to generate 30 components, with varFeatures = 50,000. For visualization, we used the addUMAP function in ArchR with the following parameters, nNeighbors = 100; minDist = 0.05; metric = cosine; and corCutOff = 0.35. Clustering was performed using the addClusters function in ArchR with the following parameters, method = ‘Seurat’; knnAssign = 100; and corCutOff = 0.35 and maxClusters = 14 to match the number of clusters identified in the scRNA-seq data. We then used the addHarmony function in ArchR to correct for batch effects. After batch-effect correction, we reperformed clustering and visualization using the same parameters.

#### Peak-calling and TF motif accessibility scoring

Filtered fragments for cells in each sample were aggregated and used as input to the MACS2 peak caller^50^; parameters -f BED, -g 2.7e9,–no-model,–shift -75,–extsize 150, -q 0.05). Peaks are filtered using an IDR cutoff of 0.05. We subsequently added motif annotations using ‘addMotifAnnotations’ with the CisBP motif database and computed chromVAR deviations for each single cell with ‘addDeviationsMatrix’^24^.

#### Identifying enriched motifs per cluster

To identify differentially accessible motifs for each group of interest, we used the rank_genes_groups function in scanpy with method = ‘wilcoxon’ and corr_method = ‘benjamini-hochberg’ on the chromVAR zscore matrix. Motifs were filtered to include only those for which the corresponding TF was expressed by >20% of cells in the corresponding FLS state. The top-six motifs after ranking by ‘score’ for each state were then selected for plotting in the heat map in Fig. 3d.

### In vitro FLS culture and stimulation for bulk RNA sequencing

Synovial tissues from four donors (RF^+^ and/or CCP^+^) were disaggregated and cultured as above. At passage 4 or 5, cells from the four donors were pooled and were plated into 12-well plates at 70,000 cells per well. Cells were allowed to adhere and were then stimulated with TNF (0.1 ng ml^−1^), IFN-γ (0.05 ng ml^−1^) or IL-1β (0.01 ng ml^−1^) alone or in combination for 24 h in triplicate. For DLL4 treatment, cell culture plates were coated with 0.5 μg ml^−1^ recombinant DLL4-Fc (R&D systems) overnight at 4 °C before the addition of FLS. Concentrations for all stimuli were determined based on an initial titration experiment with four concentrations per stimulus (10× dilutions starting with 100 ng ml^−1^ TNF, 50 ng ml^−1^ IFN-γ, 10 ng ml^−1^ IL-1β and 5 μg ml^−1^ DLL4-Fc) and performed bulk 3′ RNA sequencing to determine approximate midpoints on dose–response curves for the majority of DEGs. To stay within the dynamic range of gene expression, we used the lowest concentrations where we still obtained ~1,000 significantly DEGs. After stimulation, we lysed cells, isolated RNA (Zymo Research R1052), prepared libraries (Lexogen QuantSeq 3′ mRNA-Seq Library Prep kit (FWD) for Illumina 015.96) and the IGO at the Sloan Kettering Institute sequenced samples (bulk RNA sequencing).

### Stimulated FLS bulk RNA sequencing data analysis

Reads from 3′ RNA sequencing of fibroblasts treated with cytokines were processed using v.2.5.3 of the snakePipes mRNA-seq pipeline^51^ using the flags –reads ‘_R1_001’ ‘_R2_001’–mode ‘alignment’–trim–trimmerOptions ‘-a A{10}N{90}’. In brief, this pipeline trims reads using Cutadapt, aligns them using STAR to the genome (release 34 of GRCh38 with Gencode annotations) and then aggregates gene-level counts using featureCounts. DEGs for each condition were then defined relative to control cells using DESeq2. Genes that were up- or downregulated at *P* < 0.05 following correction for multiple hypothesis testing for each single cytokine treatment were used to define expression signatures for each cytokine. The distributions shown in Fig. 4c are of the (shrunken) log_2_ fold change estimates of these genes relative to control cells estimated by DESeq2 in cells treated with the indicated cytokines or combinations of cytokines.

### Flow cytometry of stimulated cultured FLS

FLS derived from six different RA synovial tissues were cultured until passage 4–5. Each primary FLS line was split into six wells and stimulated with TNF (20 ng ml^−1^), IFN-γ (100 U ml^−1^), IL-1β (1 ng ml^−1^) or combinations thereof for 72 h before collection for flow cytometric measurement of surface protein expression (BD FACSymphony A3). Data analysis via FlowJo v.10.8.1.

### Multicolor IF

Synovial tissue was fixed in 1:4 dilution Fixation/Permeabilization solution (BD Biosciences Cytofix/Cytoperm cat no. 554714) in PBS for 16–20 h at 4 °C. Tissue was washed 3× then placed in 30% sucrose in 0.1 M sodium phosphate buffer pH 7.4 until the tissue sank, at which point it was embedded in optimal cutting temperature compound (OCT), frozen on dry ice and stored at −80 °C until sectioning (10-μm thick). For staining, tissues were rehydrated on slides, permeabilized with 0.1% Triton and blocked with 5% normal goat serum (Thermo Fisher Scientific, 31873) before staining with primary antibodies (5 h at room temperature or 21 h at 4 °C) followed by secondary antibodies (2 h room temperature). Appropriate isotype controls were used on a separate section. After antibody stains, slides were washed, stained for nuclei (DAPI, Thermo Fisher Scientific, 62248; 1:2,000 for 5 min room temperature) and mounted with Fluoromount G (Thermo Fisher, 00-4958-02). Images were acquired with a Leica SP8 confocal microscope (40x oil immersion). Image analysis, including segmentation and intensity measurements, was performed with Imaris cell-imaging software.

### Stimulation of sorted FLS

RA synovial tissues from five donors were disaggregated as above, stained with antibodies to CD45 (2D1), CD31 (WM59), PDPN (NZ-1.3), THY1 (5E10), CD34 (581) and Zombie NIR (BioLegend) for fluorescence-activated cell sorting (BD FACSAria III Cell Sorter). FLS populations (CD45^−^CD31^−^PDPN^+^THY1^−^CD34^−^ versus CD45^−^CD31^−^PDPN^+^CD34^+^) were collected directly into cell culture medium and plated at 20,000 cells per well in a 96-well plate. After adhering for 30 min, they were stimulated for 24 h with TNF (20 ng ml^−1^), IFN-γ (100 U ml^−1^) and IL-1β (1 ng ml^−1^) or a medium-only control. Supernatant was collected, centrifuged and assayed via a custom ProcartaPlex assay (Thermo Fisher Scientific) on a Luminex 200 Instrument. The cells were collected in trizol for bulk RNA sequencing.

### Cultured FLS IF

Cultured FLS from four RA synovial tissues were plated on glass plates coated with 0.1 mg ml^−1^ poly-l-lysine in PBS for 5 min at 37 °C with or without subsequent coating with 5 μg ml^−1^ DLL4-Fc (R&D systems) overnight at 4 °C. After adhering for 3 h, FLS were stimulated with IFN-γ (1 U ml^−1^) or IL-1β (0.01 ng ml^−1^) for 1 h followed by immediate fixation with 4% PFA for 10 min, permeabilization with 0.1% triton and staining with primary and secondary antibodies and DAPI as above in section on multicolor IF.

### Spatial transcriptomics

Fresh synovium was immediately embedded in OCT and frozen using isopentane cooled by liquid nitrogen. We used Visium Spatial Gene Expression platform (10x Genomics) in conjunction with the IGO and Molecular Cytology core facilities at the Sloan Kettering Institute. For this, tissue was sectioned (10-μm sections, two tissue sections in two replicates each per slide, capture area 6.5 × 6.5 mm), stained with H&E, permeabilized at 37 °C for 12 min and polyadenylated mRNA was captured by primers bound to the slide. Reverse transcription, second strand synthesis, cDNA amplification and library preparation proceeded using the Visium Spatial Gene Expression Slide & Reagent kit (10x Genomics PN 1000184) according to the manufacturer’s protocol. After evaluation by real-time PCR, sequencing libraries were prepared with 14 cycles of PCR. Indexed libraries were pooled equimolar and sequenced on a NovaSeq 6000 in a PE28/120 run using the NovaSeq 6000 SP Reagent kit (200 cycles) (Illumina). An average of 170 million paired reads were generated per sample.

### Spatial transcriptomics data analysis

#### Preprocessing and dimensionality reduction

Spatial sequencing data from two tissues (two sections each) were aligned using the Space Ranger (v.1.2.2, 10x Genomics) pipeline to the reference genome refdata-gex-GRCh38-2020-A using default parameters to derive a feature spot-barcode gene expression matrix. Combining the four sections yielded a filtered count matrix of 12,257 spots by 19,809 genes, with a median of 1,754 molecules per spot. The count matrix was then normalized by library size and scaled to the median of total counts of all cells before normalization for analysis of the combined dataset. We then natural-log-transformed the reads with a pseudocount of 1. Seurat v.3.2 package was then used to select top variable genes for spatial RNA-seq clustering. Highly variable genes were identified using the scanpy highly_variable_genes function with batch_key = ‘sample’ and n_top_genes = 2,000. PCA was performed on the normalized expression of highly variable genes, with the top 50 PCs retained.

#### Topic modeling

To characterize cell types in and investigate the relationships between the Louvain clusters using topic modeling, Latent Dirichlet Allocation models were trained on individual ST datasets using the R package CountClust v.1.18.011,12 (refs. ^52,53^). Genes that were detected in fewer than ten spots or greater than 95% of the spots were removed and topic models were fitted to the raw gene counts. The number of topics, K, was selected based on the maximum Bayes factor value. The matrix containing the posterior probability of each topic across every spot, ω, was used to visualize the prevalence of each topic in each spot cluster; the spot clusters were generated using the R package Seurat v.3 (ref. ^54^). The top-30 genes for each topic were chosen by identifying the genes that best discriminate the topic from all other topics using a Kullback–Leibler (KL) divergence-based score as implemented in CountClust (treating the gene’s probability in the topic as the mean of a Poisson distribution)^53^. Pathway analysis was conducted using the R package GProfiler v.0.2.113 of these top-30 genes in each topic^55^.

#### Scoring gene signature expressions

To score the single-cell expression of gene signatures, we further transformed the data by *z* score and calculated the average expression of each curated gene set per cell type subtracted from the average expression of a reference set of genes using the score_genes function in scanpy. The subsequent cell type scores were transformed again by *z* score. Gene signature expressions were visualized using the scanpy.pl.spatial function.

### Reporting summary

Further information on research design is available in the Nature Portfolio Reporting Summary linked to this article.

## Online content

Any methods, additional references, Nature Portfolio reporting summaries, source data, extended data, supplementary information, acknowledgements, peer review information; details of author contributions and competing interests; and statements of data and code availability are available at 10.1038/s41590-023-01527-9.

## Supplementary information

Reporting Summary

Supplementary Tables Supplementary Table 1. Patient characteristics for RA synovial tissue samples used in this study. Supplementary Table 2. DEGs for clusters defined in Fig. 1. Supplementary Table 3. GSEA results (Hallmark, FDR < 0.25 shown with Benjamini–Hochberg-adjusted p-values reported) for each cluster and FLS state. Supplementary Table 4. DEGs for states defined in Fig. 1. Supplementary Table 5. Differentially accessible motifs (chromVAR) for FLS states and scRNA- seq clusters. Includes all motifs among the top 20 most differentially accessible across clusters/states. Supplementary Table 6. Positively differentially expressed genes for RA FLS stimulated with cytokines in vitro. Supplementary Table 7. Genes differentially expressed with the addition of Notch ligand DLL4 to cytokine stimulation. *P* values were computed using a Wald test and corrected for multiple testing using the Benjamini–Hochberg procedure as implemented in the software package DESeq2. Supplementary Table 8. Defining genes (top 30) for each topic shown in Fig. 5d,e. Supplementary Table 9. List of antibodies used in this study.

## Data Availability

The data supporting this publication have been deposited at ImmPort (https://www.immport.org) under study accession SDY2213. An h5ad file for CELLXGENE interactive data viewer is also available for download. Source data are provided with this paper.

## Source data

Source Data Fig. 4 Source data for graph.
Source Data Extended Data Fig. 4 Source data for bar graphs.
Source Data Extended Data Fig. 5 Source data for bar graphs.

## Code availability

The customized code used in the present study is publicly available at: https://github.com/viannegao/RA_Fibroblast_Multiome_Analysis.git
