# Supplementary material for: Drivers of heterogeneity in synovial fibroblasts in rheumatoid arthritis
Source: Nat Immunol. 2023 Jun 5;24(7):1200–10. doi: 10.1038/s41590-023-01527-9 (PMC10307631; doi:10.1038/s41590-023-01527-9)
Supplement: Supplementary file 1 — Reporting Summary [file 41590_2023_1527_MOESM1_ESM.pdf]

Corresponding author(s): Rudensky, Alexander Y
Leslie, Christina S
Smith, Melanie H

Last updated by author(s): April 26, 2023

Reporting Summary

Nature Research wishes to improve the reproducibility of the work that we publish. This form provides structure for consistency and transparency in reporting. For further information on Nature Research policies, see our Editorial Policies and the Editorial Policy Checklist.

Statistics

For all statistical analyses, confirm that the following items are present in the figure legend, table legend, main text, or Methods section.

n/a	Confirmed	
☐	☒	The exact sample size (*n*) for each experimental group/condition, given as a discrete number and unit of measurement
☐	☒	A statement on whether measurements were taken from distinct samples or whether the same sample was measured repeatedly
☐	☒	The statistical test(s) used AND whether they are one- or two-sided<br>*Only common tests should be described solely by name; describe more complex techniques in the Methods section.*
☒	☐	A description of all covariates tested
☒	☐	A description of any assumptions or corrections, such as tests of normality and adjustment for multiple comparisons
☐	☒	A full description of the statistical parameters including central tendency (e.g. means) or other basic estimates (e.g. regression coefficient) AND variation (e.g. standard deviation) or associated estimates of uncertainty (e.g. confidence intervals)
☐	☒	For null hypothesis testing, the test statistic (e.g. *F*, *t*, *r*) with confidence intervals, effect sizes, degrees of freedom and *P* value noted<br>*Give P values as exact values whenever suitable.*
☒	☐	For Bayesian analysis, information on the choice of priors and Markov chain Monte Carlo settings
☒	☐	For hierarchical and complex designs, identification of the appropriate level for tests and full reporting of outcomes
☐	☒	Estimates of effect sizes (e.g. Cohen's *d*, Pearson's *r*), indicating how they were calculated

Our web collection on statistics for biologists contains articles on many of the points above.

Software and code

Policy information about availability of computer code

Data collection: No software was used for data collection.

Data analysis: The customized code used in the present study is publicly available at: https://github.com/viannegao/RA_Fibroblast_Multiome_Analysis.git
Data analysis software used:
FlowJo v10.8.1
Prism v9.4.1
Imaris v9.6.1 / v9.7.1
ArchR v1.0.1
Bowtie2 v2.4.1
cellranger-arc v1.0.1
ChromVAR v1.20.2
CountClust v1.18.011
DESeq2 v1.38.3
DoubletDetection v4.1
fastq-dump v3.0.3
fGSEA v1.24.0
GProfiler v0.2.113 R
MACS2 v2.2.7.1
MAST v1.8.2
Palantir v1.0.0
PhenoGraph v1.5.7
SAMtools v1.8
scanpy v1.7.3

Space Ranger v1.2.2
SnakePipes v2.5.3

For manuscripts utilizing custom algorithms or software that are central to the research but not yet described in published literature, software must be made available to editors and reviewers. We strongly encourage code deposition in a community repository (e.g. GitHub). See the Nature Research guidelines for submitting code & software for further information.

Data

Policy information about availability of data

All manuscripts must include a data availability statement. This statement should provide the following information, where applicable:

- Accession codes, unique identifiers, or web links for publicly available datasets
- A list of figures that have associated raw data
- A description of any restrictions on data availability

The data supporting this publication have been deposited at ImmPort (https://www.immport.org) under study accession SDY2213 (accessible with next release scheduled May 26, 2023). An interactive data viewer is also available for download.

Field-specific reporting

Please select the one below that is the best fit for your research. If you are not sure, read the appropriate sections before making your selection.

☒ Life sciences ☐ Behavioural & social sciences ☐ Ecological, evolutionary & environmental sciences

For a reference copy of the document with all sections, see nature.com/documents/nr-reporting-summary-flat.pdf

Life sciences study design

All studies must disclose on these points even when the disclosure is negative.

Sample size	No sample size calculation was performed. Based on published scRNAseq data from the synovium, we estimated that we could isolate sufficient FLS to detect multiple inflammation associated FLS clusters from 2 highly inflamed synovia. We then sequenced an additional 3 samples with varying degrees of leukocyte infiltration.
Data exclusions	Sequencing data for cells that did not past quality control was excluded from downstream analysis. See Methods section "Pre-processing of single cell multiome ATAC + gene expression data" for details.
Replication	Multiome and spatial transcriptomics were replicated with five separate RA patient synovial samples.
Randomization	Not performed as there was no intervention.
Blinding	Not performed as there was no intervention.

Reporting for specific materials, systems and methods

We require information from authors about some types of materials, experimental systems and methods used in many studies. Here, indicate whether each material, system or method listed is relevant to your study. If you are not sure if a list item applies to your research, read the appropriate section before selecting a response.

Materials & experimental systems

n/a	Involved in the study
☐	☒ Antibodies
☐	☒ Eukaryotic cell lines
☒	☐ Palaeontology and archaeology
☒	☐ Animals and other organisms
☐	☒ Human research participants
☒	☐ Clinical data
☒	☐ Dual use research of concern

Methods

n/a	Involved in the study
☒	☐ ChIP-seq
☐	☒ Flow cytometry
☒	☐ MRI-based neuroimaging

Antibodies

Antibodies used	For sorting FLS:<br>Anti-CD45-FITC (eBiosciences; 11-9459-42, 2D1; lot 4271593; 1:100)<br>Anti-PDPN-APC (Invitrogen; 17-9381-42; NZ-1.3; lot 1988690; 1:100)

Anti-CD31- PE/Cy7 (Biolegend; 303118; WM59; lot B276836; 1:100)
Anti-CD90/THY1-BV650 (Biolegend, 328144, 5E10, lot B362535, 1:100)
Anti-CD34- PE (Biolegend, 343506, 581, lot B351594, 1:100)
Ghost Dye Violet 510 (Tonbo; 13-0870-T100; no clone; lot D0870040521133; 1:1000)

For flow cytometry of cultured FLS:
Anti-PDPN-APC (Invitrogen; 17-9381-42; NZ-1.3; lot 1988690; 1:200)
Anti-CD90/THY1-BV650 (Biolegend, 328144, 5E10, lot B362535, 1:100)
Anti-HLA.DR- FITC (Biolegend; 307604, L243, lot B275368; 1:50)

For immunofluorescence:
Primary:
Anti-PDPN (Invitrogen; 14-9381-82; NZ-1.3; lot 2400405; 1:100 – final 5 ug/mL)
Anti-HLA.DR-AF488 (Biolegend; 307620; L243; lot B271228; 1:100 – 2 ug/mL)
Anti-CD3-BV480 (BD biosciences; 566105; UCHT1; lot 0079903; 1:100)
Anti-CD8-AF647 (Biolegend; 344725; SK1; lot B270006; 1:50 – final 1 ug/mL)
Anti-pSTAT1-PE (Biolegend; 686403; A15158B; lot B327686; 1:50 – final 0.12 ug/mL)
Anti-cJun (Cell Signaling Technology; 9165T; 60A8, lot 13; 1:250)
Anti-CD68-AF488 (Biolegend; 333812; Y1/82A; lot B278908; 1:10 – final 2.4 ug/mL)
Anti-CD163-AF647 (Biolegend; 333619; GH1/61; lot B353001; 1:100 – final 1.5 ug/mL)
Anti-CD19-PE (Biolegend; 302208; HIB19; lot B273506; 1:20 – final 2.5 ug/mL)
Anti-CD90/THY1-AF700 (R&D systems; FAB2067N; Thy1A1; lot 1569061; 1:50 – final 4 ug/mL)
Anti-CD34-AF647 (Biolegend; 343507; 581; lot B312791; 1:100 – final 2 ug/mL)
Anti-CD31-AF488 (Biolegend; 303109; WM59; lot B290397; 1:50 – final 4 ug/mL)

Secondary:
Anti-rat-AF594 (Biolegend; 405422; polyclonal; lot B302011; 1:1000)
Anti-rabbit-AF488 (Thermo Fischer; A-11034; polyclonal; lot 1737902, 1:1000)

Validation

For flow cytometry / sorting FLS:
Anti-CD45-FITC (2D1) -- Mizoguchi et al (see main references)
Anti-PDPN-APC (NZ-1.3) -- Mizoguchi et al
Anti-CD31- PE/Cy7 (WM59) -- Mizoguchi et al
Anti-CD90/THY1-BV650 (5E10) -- same IF sigal as glone Thy1A1 used in Mizoguchi et al
Anti-CD34- PE (581) -- same IF signal as clone EP373Y used in Mizoguchi et al (preferred the clone 581 as it came in a fluorophore conjugated format)
Anti-HLA.DR- FITC (L243) -- Radtke et al

For immunofluorescence:
Anti-PDPN (NZ-1.3) -- Mizoguchi et al
Anti-HLA.DR-AF488 (L243) -- Radtke et al
Anti-CD3-BV480 (UCHT1) -- Radtke et al
Anti-CD8-AF647 (SK1) -- Radtke et al
Anti-pSTAT1-PE (A15158B) -- Lin et al
Anti-cJun (60A8) -- Larsen et al.
Anti-CD68-AF488 (Y1/82A) -- Lin et al
Anti-CD163-AF647 (GH1/61) -- Radtke et al
Anti-CD19-PE (HIB19) -- Alivernini et al and Zhang et al (see main references)
Anti-CD90-AF700 (Thy1A1) -- Mizoguchi et al
Anti-CD34-AF647 (581) -- same signal as clone EP373Y used in Mizoguchi et al (preferred the clone 581 as it came in a fluorophore conjugated format)
Anti-CD31-AF488 (WM59) -- Radtke et al

References (not in primary references for the manuscript)

Larsen SB, et al. Establishment, maintenance, and recall of inflammatory memory. Cell Stem Cell. 2021 Oct 7;28(10):1758-1774.e8. doi: 10.1016/j.stem.2021.07.001. Epub 2021 Jul 27.

Lin JR, et al. Highly multiplexed immunofluorescence imaging of human tissues and tumors using t-CyCIF and conventional optical microscopes. Elife. 2018 Jul 11;7:e31657. doi: 10.7554/eLife.31657.

Radtke AJ, et al. IBEX: an iterative immunolabeling and chemical bleaching method for high-content imaging of diverse tissues. Nat Protoc. 2022 Feb;17(2):378-401. doi: 10.1038/s41596-021-00644-9. Epub 2022 Jan 12.

Eukaryotic cell lines

Policy information about cell lines

Cell line source(s)	Only primary synovial fibroblast cell lines were used (no established cell lines).
Authentication	n/a
Mycoplasma contamination	cells were not tested for mycoplasma

Commonly misidentified lines (See ICLAC register)	n/a

Human research participants

Policy information about studies involving human research participants

Population characteristics	See supplemental table 1.
Recruitment	Under IRB 2014-233, all patients <18 years of age with rheumatoid arthritis (RA) satisfying the 1987 and/or 2010 ACR/EULAR classification criteria and undergoing arthroplasty or synovectomy at the Hospital for Special Surgery main campus were identified via an electronic medical record screen. The charts of patients identified via the electronic medical record screen were reviewed by a board certified rheumatologist to assess the likelihood of a true diagnosis of RA based of diagnoses, lab results, x-rays and active medications. For those patients with a high likelihood of having RA and with the approval of their surgeons, patients were contacted by a research assistant and invited to participate in the study. Patients were enrolled preoperatively and information regarding demographics, medical history and disease activity was collected. Informed consent was obtained from all participants. Participates were not compensated for their involvement in the study.
Ethics oversight	Patient samples were collected under the approval of Hospital for Special Surgery IRB 2014 -233 and Memorial Sloan Kettering Cancer Center IRB 06-107.

Note that full information on the approval of the study protocol must also be provided in the manuscript.

Flow Cytometry

Plots

Confirm that:

☒ The axis labels state the marker and fluorochrome used (e.g. CD4-FITC).

☒ The axis scales are clearly visible. Include numbers along axes only for bottom left plot of group (a 'group' is an analysis of identical markers).

☒ All plots are contour plots with outliers or pseudocolor plots.

☒ A numerical value for number of cells or percentage (with statistics) is provided.

Methodology

Sample preparation	Synovial tissue samples were disaggregated into a single-cell suspension using published methods. Briefly, fragments were minced and enzymatically digested (Liberase TL (Sigma-Aldrich) 100 ug/mL and DNaseI (New England Biolabs) 100 µg/mL in RPMI) for 30 min at 37°C. Disaggregated cells were assessed for quality and viability (Nexcelom Cellometer Auto 2000) and then stained with antibodies to CD45 (2D1), CD31 (WM59), PDPN (NZ-1.3) and Ghost Dye Violet 510 (Tonbo) for fluorescence activated cell sorting (BD FACSAria III Cell Sorter).
Instrument	BD FACSAria III Cell Sorter
Software	FlowJo 10.8.1
Cell population abundance	Post sort fractions can be found in Extended Data Figures 1 and 4 along with gating strategy. The purity of samples was not independently verified.
Gating strategy	single cells, live cells, CD45-, CD31-, PDPN+.<br>For Extended Data Figure 4, cells were further sorted into THY1-, CD34- versus CD34+<br>See Extended Data Figure 1 and Extended Data Figure 4 for gates.

☒ Tick this box to confirm that a figure exemplifying the gating strategy is provided in the Supplementary Information.
